# Bridging the educational gaps of health professionals in oncogenomics: results from a pilot e-learning course

**DOI:** 10.3389/fmed.2024.1422163

**Published:** 2024-11-21

**Authors:** Emilia Stellacci, Simone Martinelli, Pietro Carbone, Elena Demuru, Maurizio Genuardi, Paola Ghiorzo, Giuseppe Novelli, Alessandra Di Pucchio, Federica Maria Regini, Debora Guerrera, Andrea Vittozzi, Donatella Barbina, Serenella Venanzi, Marc van den Bulcke, Stefania Boccia, Alfonso Mazzaccara, Arcangela De Nicolo, Roberta De Angelis

**Affiliations:** ^1^Department of Oncology and Molecular Medicine, Istituto Superiore di Sanità, Rome, Italy; ^2^Training Service, Istituto Superiore di Sanità, Rome, Italy; ^3^Department of Life Sciences and Public Health, Università Cattolica del Sacro Cuore, Rome, Italy; ^4^Medical Genetics Unit, Fondazione Policlinico Universitario A. Gemelli IRCCS, Rome, Italy; ^5^Department of Internal Medicine and Medical Specialties, University of Genoa, Genoa, Italy; ^6^IRCCS Ospedale Policlinico San Martino, Genetics of Rare Cancers, Genoa, Italy; ^7^Department of Biomedicine and Prevention, Genetics Section, University of Rome “Tor Vergata”, Rome, Italy; ^8^Cancer Centre Epidemiology and Public Health, Sciensano, Brussels, Belgium; ^9^Section of Hygiene, University Department of Life Sciences and Public Health, Università Cattolica del Sacro Cuore, Rome, Italy; ^10^Center for Omics Sciences, IRCSS San Raffaele Scientific Institute, Milan, Italy

**Keywords:** distance learning, genetic/medical education, oncogenomic literacy, problem-based learning, personalized medicine, health professionals

## Abstract

**Background:**

Genetic and genomic literacy of health professionals is of utmost importance to realize the full potential of personalized medicine. As part of a European Union project, we piloted an e-learning course on oncogenomics, primarily targeted to physicians, and we assessed both its effectiveness and users’ satisfaction.

**Methods:**

The course materials were developed in English according to the Problem-Based Learning method. Learning objectives, covering the basic principles of genetics and the OMICS technologies applied to oncology, were defined based on previously identified core competencies. We used a pre-test vs. post-test study design to assess knowledge improvements. Performance results by demographic and professional characteristics of participants were analyzed using univariate or multivariate statistical methods.

**Results:**

Overall, 346 Italian professionals (61% physicians, 39% biologists) successfully completed the course. Their average post-test score was almost 19% higher than the pre-test (71.6% vs. 52.9%), with no significant differences by sex. Older age (>50 years) and southern area of residence were both correlated with higher gains. The average proportion of correct answers in the *final certification test* after three attempts was 85% (69% at first attempt), with some differences across professional categories. Methodology, quality of content and usability of the e-learning platform were all highly rated via satisfaction questionnaire (average scores between 4 and 5, scale 1 to 5).

**Conclusion:**

The pilot phase confirmed the suitability of the e-learning as a cost-effective method to improve oncogenomic literacy of health professionals. Translation into natural languages and accreditation by European or country-specific Continuing Medical Education systems will be the main incentives for wider dissemination.

## Highlights

- A properly trained health care workforce is essential to realize the full potential of personalized medicine for cancer prevention and treatment.
- We developed a distance e-learning course on oncogenomics for health professionals starting from a curriculum of knowledge and attitudes identified based on literature and expert consensus.
- The pilot course proved effective in significantly increasing knowledge and was very positively evaluated with regards to content and training modality.
- The use of the native language and the recognition of credits for continuing medical education are relevant incentives to be considered for the systematic implementation of similar training initiatives.

## Introduction

The advent and rapid implementation of massive parallel sequencing (also known as next generation sequencing, NGS) technologies brought about a revolution in genetic and genomic research holding the promise to transform the entire spectrum of disease management, from risk assessment to diagnosis, prognosis, and treatment (1, 2). To translate the great potential into real benefit and move genomic medicine forward, several hurdles should be overcome, requiring substantial changes in the system and infrastructure, which include improved literacy of professionals, citizens, and decision makers on genetic and genomic matters (3–6).

Gaps in the genetic and genomic education of health care providers have long been appreciated as a roadblock to the implementation of research discoveries into clinical practice (7–12). In a rapidly evolving scenario, the development (and maintenance) of a competent workforce has become a prerequisite for bridging the research and clinical settings. Genetic and genomic literacy of health professionals is of utmost relevance to the oncology field. Cancer diagnosis and treatment are becoming increasingly molecular-based, thanks to the decreasing costs of sequencing technologies and the growing knowledge of the human genome. The widespread use of NGS techniques offers increased opportunities to tailor cancer prevention and treatment, through targeted agents and immunotherapies. This new scenario requires responsible interpretation, communication, and application of germline and somatic test results for informed cancer prevention and care, and improved health outcomes (13–16).

Bridging the educational gaps in oncogenomics of non-genetic health professionals was one of the objectives of the Innovative Partnership for Action Against Cancer (iPAAC), a Joint Action (JA) co-funded by the European Commission and 24 Member States, aimed at developing innovative approaches to advance all dimensions of cancer control.^1^ Within this framework, a set of core competencies in cancer genomics for non-genetic health professionals was defined (17). On this ground, we set out to develop and pilot an e-learning course aimed at improving knowledge and attitude of health professionals on the fundamentals of genetics and on the main applications of genomic technologies in clinical oncology. For this purpose, we exploited the expertise in distance learning methodologies available at the Italian National Institute of Health (Istituto Superiore di Sanità, ISS). The exponential surge of e-learning courses during the COVID-19 pandemic and post-COVID era has further propelled virtual learning platforms to the forefront of continuous education and upskilling for healthcare professionals (18–20).

We herein describe the stepwise strategy we employed to design and test the *Oncogenomics for Health Professionals* e-learning course, we report the results of the pilot phase performed in Italy (including training effectivity and users’ satisfaction), and we elaborate on the feedback received from the participants to delineate further implementation steps.

## Methods

### Course design

The course was structured in four specific *Learning Objectives* (LOs). The content of the LOs was built starting from 37 core competencies in cancer genomics required for non-genetic physicians, which were defined via a two-step consensus-based approach, i.e., first identification via systematic literature review and then refinement by an international expert panel, based on a modified Delphi method (17). The original set of core competencies pertained to the *Knowledge*, *Attitudes* and *Abilities* domains. Because of the training modality adopted in the e-learning course, we have retained all the competencies related to the *Knowledge* domain and a few competencies from the *Attitudes* domain, which can be conveniently transferred and measured in a low interaction e-learning setting, while we have not selected any from the *Abilities* domain because they require an interactive setting.

All course materials were developed in English to facilitate dissemination across European countries, after a pilot phase implemented in Italy. The course was accredited by the Italian provider of Continuing Medical Education (CME), Age.na.s. (Agenzia nazionale per i servizi sanitari regionali), for a total of 16 h credits.

### Learning methodology

The course was developed according to the main models of andragogic training, specifically to the Problem-based Learning (PBL) method – a training methodology that encourages the participants to “learn to learn” by solving real-world problems that reflect their professional field (21, 22).

In the PBL method, the starting point of the training process is the Problem, which prompts the participants to reflect on their professional experience and knowledge and to identify their learning needs relevant to the course learning objectives. The questions and keywords in the Problem guide the learning process toward practical implications in the participants’ professional field. In this way, the participants are actively involved and encouraged to expand their knowledge and to acquire new Problem-solving skills, studying the training material selected by the experts and carrying out additional, independent research.

The Learning Management System (LSM) used to develop the e-learning was the Totara Learn 11, which is based on a Moodle extension and offers all technical resources to implement the PBL methodology. Low interaction, asynchronous mode, and no facilitation were the main technical features chosen.

The PBL methodology was set up using platform tools such as feedback, web pages, quizzes and other learning tools (e.g., the Shareable Content Object Reference Model, SCORM). The e-learning path was scheduled in sequential steps, whereby access to the activities in each unit was allowed only when those of the previous one were completed.

The PBL cycle can be described using a variety of resources. The process begins with a scenario introduction, presenting the central challenge or problem. Participants then engage in a critical analysis activity, often utilizing a SCORM-based tool, to dissect the problem and define specific learning objectives. This is followed by an independent exploration phase, where participants leverage a range of resources: bibliographic references, curated website lists, in-depth reading materials, and expert video tutorials. Finally, participants apply their acquired knowledge and skills to think about a solution to the original problem.

### Evaluation tools

The design of the e-learning course included the three following assessment tools:

- *Self-assessment test*: This mandatory test included 12 Multiple Choice Questions (MCQs), three for each LO, offered both at the entry (T0, pre-test) and at the end of the course (T1, post-test), before the *final certification test*. This test allowed the participants to assess their own level of knowledge. For each question, there were four possible answers, only one of which was correct. No time limit was set for this test, and a minimal score was not required. At T0, participants were allowed only one attempt and did not receive a score, but only feedback on the correctness of their answers and possibly a suggestion on where to find the correct information during the training. After completing this initial test, learners gained access the training unit package. At T1, participants could make multiple attempts, allowing them to repeat the test until they felt adequately prepared for the *final certification test*. At this step, participants received feedback on their wrong answers, directing them to the specific learning materials to review before attempting the *certification test*. Because both T0 and T1 are self-assessment tests of learning, the time between attempts was up to the participant’s individual choice.
- *Final certification test*: This mandatory test was administered at the end of the course, enabling the participants to earn CME credits. A minimum of 75% correct answers was required to pass this test. Three hours (and a maximum of three attempts) were allowed to complete the test, which included 48 MCQs, 12 for each LO. The participants, based on the score and the need for further studies, established the time interval between attempts.
- *Satisfaction questionnaire*: This test was mandatory and was proposed at the very end of the course to participants who completed the *certification test*. It allowed participants to evaluate different aspects of the e-learning process, through a standard battery of 18 closed Likert-type questions (from 1-minimum to 5-maximum level of agreement), including seven about the perceived quality of the methodology, eight about the educational contents of the course, and three about the operability of the platform. Two further open questions about the strengths of the course and suggestions to improve it completed the battery. No time limits was set for completion.

### Pilot setting

The course was open to two categories of health professionals: physicians and biologists. Primary care physicians, residents, physicians of all specialties, and biologists working in the public and private Health Service were all admitted to the course. Biologists in the Italian National Health Service play essential complementary roles to those performed by clinicians, contributing to the diagnosis, prevention and treatment of diseases. Specifically, biologists perform laboratory diagnostics (clinical analyses, clinical microbiology, genetic and molecular biology tests) and histological analyses (cytology and histopathology, cytogenetics). They are involved in prevention services (epidemiological surveillance, prevention of hospital infections), biomedical research and in conducting clinical trials. The course was aimed at promoting basic knowledge about the main applications of oncogenomics and all the addressed health professionals might require and benefit from acquaintance with genetics/genomics matters in their daily practice. The course was also attended by medical geneticists and this category was used as a “benchmark” to analyze the results scored by the other professional categories.

The e-learning course was piloted in Italy to assess its value and use and determine the level of satisfaction of the target users. Only a very limited test was run abroad (in Greece, Malta, Luxembourg, Portugal, and Norway) as a preliminary check for the wider divulgation we envisioned. The course was delivered through the ISS e-learning platform (EDUISS^2^) from April to December 2021 in Italy (from September to December 2021 in other countries), and promoted through scientific professional societies and the EDUISS platform itself. Participation was free of charge.

Upon registration on the e-learning platform, Italian participants provided the following demographic and professional information: sex, age, region of residence, and CME discipline.

### Statistical analyses

Professionals who successfully completed the course (*Completers*) encompassed participants who completed the *self-assessment test*, passed the *final certification test* with ≥75% correct answers, and filled out the *satisfaction questionnaire*. *Non-Completers*, instead, included both the participants who enrolled in the course but did not start or complete it (*dropout*) and the participants who failed the *final certification test* (i.e., scored <75% correct answers). All statistical analyses were carried out on the sub-group of *Completers*. Analyzing data from *Completers* rather than from all learners has both advantages and limitations, which are discussed below (Discussion section). To estimate the gain after the training, we computed the number and proportion of correct answers given by the *Completers* to each of the 12 MCQs of the *self-assessment test* and used the McNemar test to compare post-test vs. pre-test overall results. Given the educational value of this test, we considered the attempt with the highest score at T1. Additionally, we compared the average scores of the post- and pre-test using a t-test for paired data. Both tests were applied to the *Completers* as a whole and after stratification, based on demographic and professional characteristics. The Anova for repeated measures was employed to ascertain whether there were any significant differences between the score gains achieved by physicians and biologists in the pre- and post-tests.

We used multivariate regression analysis to identify the characteristics associated with an increased number of correct answers between post- and pre-tests. For this purpose, we fitted a Poisson model using the number of additional correct answers in the post-tests vs. the pre-tests as a dependent variable, and sex, age, geographic area of residence, and professional category as independent variables. The results are shown as Relative Risk (RR) of a higher increase in the test score compared to the reference category (i.e., women, age ≤ 39 years, Southern area of residence, Genetics). From this analysis, 51 *Completers* with a score at T0 higher than the score at T1 had to be excluded.

The average scores obtained by the *Completers* in the *final certification test* were analyzed overall and by demographic and professional characteristics. For the analysis of the *satisfaction questionnaire*, we computed the average ratings given by the *Completers* to each domain (i.e., learning methodology, contents, and platform operability). The analyses were performed using the statistical SAS Software (SAS System for Windows, version 9.4; SAS Institute, Cary, NC, USA).

## Results

We developed the course around four LOs, covering multiple topics from the genetic basis of cancer to genetic testing, OMICS technologies, pharmacogenomics, and personalized medicine. A description of the contents of the LOs and the core competencies used to define them are reported in Table 1.

**Table 1:** Learning objectives (LOs).

Learning objectives (LOs)	Contents description	LO-related core competencies of physicians
1. To describe the basic elements of human genetics and oncogenetics and the OMICS technologies used to identify the molecular signatures of cancer	Fundamental principles of human genetics, with a focus on oncogenetics (i.e., somatically acquired or germline-transmitted changes). Basic information on currently available OMICS technologies to identify molecular cancer signatures.	**Knowledge:** Basic knowledge of genetics within own field of clinical practice Knowledge of the concept of somatic genetic changes Knowledge of the role of genomic changes in the pathophysiology and treatment of cancer Understanding the hereditary predisposition to cancer, including the polygenic and multifactorial nature of cancer risk Knowledge of the major hereditary cancer syndromes Understanding the specific characteristics of hereditary cancer syndromes that may distinguish them from sporadic cancers Understanding the differences between hereditary and non-hereditary cancer
2. To describe the currently available genetic/genomic tests for cancer screening and diagnosis, and their applications 3. To define the role of genetic testing and counseling in the risk assessment of hereditary cancers	Information on the existing genetic and genomic tests to be used for cancer screening and diagnosis, with an emphasis on the importance of genetic counseling, data interpretation, and incidental findings. Information on the role of genetic testing in assessing the possible occurrence of germline variants in cancer susceptibility genes and in estimating the risk to develop cancer based on personal and family history.	**Knowledge:** Knowledge of how genomic testing can be used to guide therapy and dose selection in cancer patients Knowledge of the availability of screening tests and procedures for those identified as having higher lifetime cancer risk Understanding genetic testing types and result interpretation Awareness of incidental and secondary findings from somatic tumour profiling Defining the general characteristic of tumour spectrum of known syndromes Awareness of overlapping phenotypes for the common syndromes that generate differential diagnosis for hereditary syndromes based on presenting cancer Knowing the interpretation and the importance of family history in assessing disease predisposition Understanding the importance of family history as a risk factor, regardless of gene testing **Attitudes:** Acknowledging the impact of genetic information on patients and their family Recognizing the need for consents to disclose a particular diagnosis to relatives Recognizing the importance of multidisciplinary work and the role of genetic counsellors as well as mental health professionals to assist patients as they process difficult information
4. To identify the main applications of pharmacogenomics and personalized medicine in oncology	Basic elements of the role and range of applications of pharmacogenomics and personalized medicine in oncology.	**Knowledge:** Knowledge of the role of genomic changes in the pathophysiology and treatment of cancer Knowledge on how genomic testing can be used to guide therapy and dose selection in patients with cancer Awareness of risk-reducing measures in high-risk patients and relatives, including chemoprevention and prophylactic surgery

Description of LOs contents and the corresponding core competencies used to develop the learning materials.

LO1 aimed at recapitulating the basic principles of human genetics, elucidating how gene variation may contribute to cancer and clarifying the difference between inherited and acquired sequence changes. Upon completion of LO1, the participants were expected to understand why cancer is a genetic disease and which OMICS technologies are most relevant for the identification of the molecular signature of cancer.

The primary aims of LO2 were to describe the genetic heterogeneity underlying cancer susceptibility and the difference between the most common genetic and genomic tests, both conventional and NGS-based, used in clinical oncology (e.g., somatic vs. constitutional testing). Emphasis was placed on understanding who should be tested for cancer predisposition and on the significance and interpretation of key terms and concepts such as variant of uncertain significance, incidental findings, and polygenic risk score.

LO3 explored the multifaceted aspects of genetic counseling when cancer predisposition is suspected to segregate within a family. The specific markers of hereditary cancer syndromes and the criteria and categories for genetic testing were debated with explanatory examples. Management strategies for individuals at high genetic risk of cancer were also covered.

Lastly, LO4 aimed at addressing why treatment is successful only in certain patients and why some individuals are more prone to adverse effects than others. To this end, the concepts of biomarkers, pharmacogenetics/pharmacogenomics, drug repositioning, and personalized medicine in oncology were extensively discussed. A relatively small number of items pertaining to the *Knowledge* domain were used to build this last LO, as all the background information needed to comprehend its specific contents had already been provided in the previous LOs.

**Figure 1:**
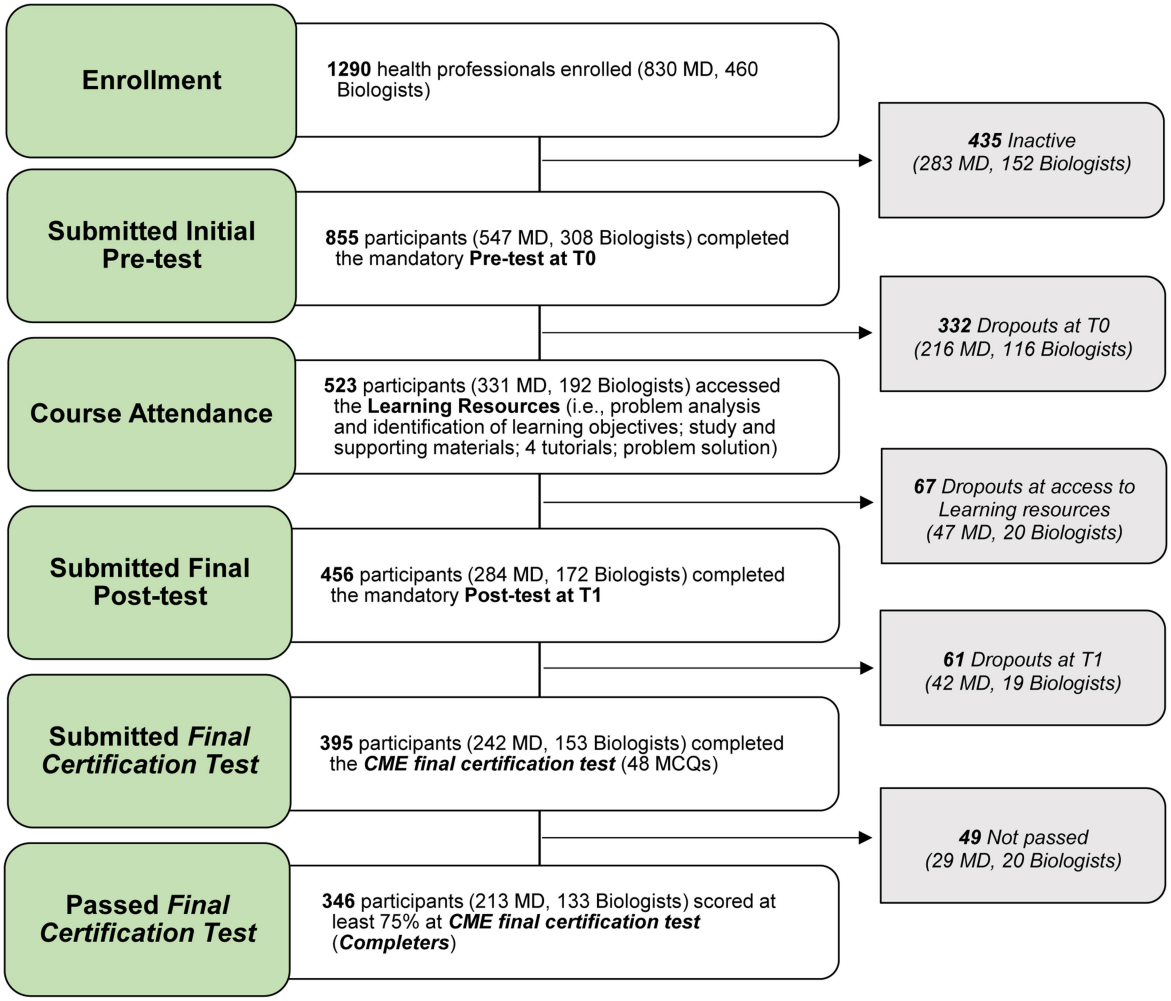
Participants’ flowchart summarizing the number of participants from the enrollment through the final certification test. MD, Medical Doctors; CME, Continuing Medical Education; MCQs, Multiple Choice Questions.

A total number of 1,290 Italian health professionals enrolled in the e-learning course. Of these, 855 completed the full set of pre-test MCQs and 456 completed the full set of post-test MCQs. Of the 395 participants who completed the *final certification test*, 346 (87.6%) obtained a score of at least 75% (so-called *Completers*). A flowchart summarizing the number of participants from the enrollment through the *final certification test* is shown in Figure 1. On average, it took 55 days for the participants to complete the course (from the date of enrollment to the date of completion of the *satisfaction questionnaire*).

Analysis of the demographic and professional characteristics of the *Completers* (Table 2) revealed a slight prevalence of women (184, 53%) vs. men (162, 47%). Median age was 49 years and the most represented age group was the youngest (≤39 years, 27%). Most participants resided in Northern Italy (39%), whereas 35 and 26% were from Southern and Central Italy, respectively. The *Completers* were mostly physicians (213, 62%), especially primary care physicians (13% of the total number of physicians who completed the course) and surgeons (10%). Physicians of other medical specialties were less represented (each <10%).

**Table 2:** Characteristics and performance of participants who completed the course (*Completers*).

*Completers*’ characteristics	Number	%	Average scores – *self-assessment test*				Average scores – *final certification test*	
			Pre-test (single attempt) (%)	Post-test (best attempt) (%)	Difference (post-test vs. pre-test) (%)	*p*-value	Best attempt	First attempt
Sex								
Men	162	47%	52.4	71.4	19.0	<0.0001	85.2	68.5
Women	184	53%	53.3	71.7	18.4	<0.0001	84.7	70.2
Age groups								
≤39	93	27%	54.4	66.7	12.3	<0.0001	83.4	70.7
40–49	81	23%	51.1	69.7	18.5	<0.0001	85.0	71.6
50–59	88	25%	53.5	76.0	22.5	<0.0001	85.8	67.2
≥60	84	24%	52.2	74.2	22.0	<0.0001	85.8	68.1
Area of residence								
North	135	39%	56.5	75.8	19.3	<0.0001	85.9	72.2
Center	91	26%	48.4	62.7	14.4	<0.0001	83.6	69.7
South	120	35%	52.2	73.5	21.4	<0.0001	84.8	66.1
Profession								
Biologist	133	38%	51.5	68.7	17.2	<0.0001	85.1	67.3
Physician	213	62%	53.7	73.4	19.6	<0.0001	84.8	70.7
Physicians’ category								
Primary care	28	13%	49.4	68.5	19.1	0.0007	85.8	74.3
Surgery	21	10%	42.5	71.0	28.6	0.0004	84.5	64.6
Public health	19	9%	47.8	73.3	25.4	0.0004	84.5	72.8
Pathology	13	6%	59.6	80.8	21.2	0.0036	85.7	67.3
Nuclear medicine	12	6%	44.5	63.9	19.4	0.0328	83.7	75.8
Residency	12	6%	66.0	82.6	16.7	0.0069	84.2	80.7
Genetics	10	5%	75.0	85.8	10.8	0.0898	88.5	84.8
Pediatrics	10	5%	57.5	74.2	16.7	0.1066	83.8	66.5
Gynecology	8	4%	60.4	76.0	15.6	0.1760	82.0	69.3
Psychiatry	8	4%	51.0	78.1	27.1	0.0481	89.9	59.6
Occupational medicine	7	3%	54.8	65.5	10.7	0.1755	82.1	67.9
Internal medicine	7	3%	59.5	66.7	7.2	0.1996	86.9	66.1
Other specialties	58	27%	54.2	73.4	19.3	<0.0001	84.2	69.2
*Final certification test* – number of attempts								
1	150	43%	57.2	74.2	17.0	<0.0001	84.2	84.2
2	145	42%	52.0	70.5	18.5	<0.0001	85.7	60.8
3	51	15%	42.7	67.0	24.3	<0.0001	84.9	50.2
Total	346	100%	52.9	71.6	18.7	<0.0001	84.9	69.4

Demographic and professional characteristics of the *Completers*. Average percentage of correct answers (average scores) obtained in the self-assessment tests and in the final certification test are displayed according to the Completers’ characteristics. Absolute differences (post- vs. pre-test) and *p*-values are shown.

The average percent of correct answers provided by the *Completers* in the *self-assessment test*, by demographic and professional characteristics, is shown in Table 2. Statistically significant improvements in average scores were generally observed, independently of the characteristics, with few exceptions. Overall, the average post-test score was almost 19 points higher than the pre-test score (71.6% vs. 52.9%, respectively). Similar increases were observed among men and women (+19% and + 18.4%, respectively). The highest increases were observed for participants older than 50 years (+22.5% points for the 50–59 year category and + 22% for the ≥60 year category) and the lowest for younger participants (+12.3%), who had the highest entry scores (54.4%). *Completers* residing in Southern and Central Italy reported the highest (+21.4%) and lowest (+14.4%) increase, respectively. A slightly higher improvement was observed for physicians (+19.6%) compared to biologists (+17.2%), but with notable differences between different medical specialties. The most remarkable statistically significant increases were observed for surgeons (+28.6%) and physicians specialized in public health (+25.4) and pathology (+21.2). As expected, based on the subjects, geneticists registered one of the smallest increases (+10.8%) and the highest average score in the pre-test (75%). Notably, participants who voluntarily withdrew after taking the pre-test (so-called *Dropouts at T0*) had an average score that was 12 points lower compared to the *Completers* (41 vs. 53).

**Figure 2:**
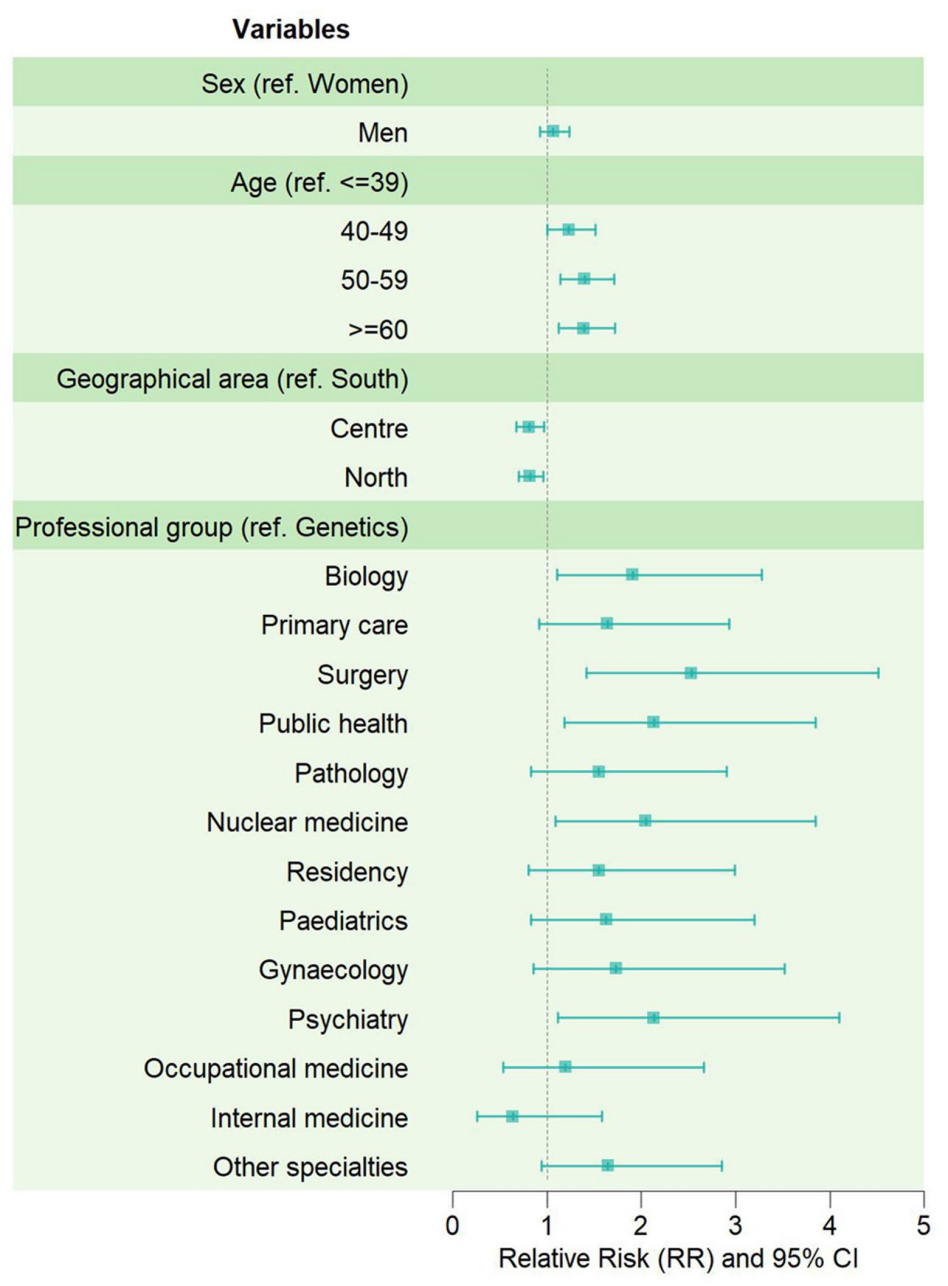
Effectiveness of the e-learning course by demographic and professional characteristics of the *Completers.* Forest-plot displaying the relative risks (RR) of getting a higher score in the post-test, compared to the pre-test, estimated through Poisson multivariate analysis.

The multivariate analysis (Figure 2) confirmed these results. Specifically, no significant differences were observed between men and women, even when adjusting for demographic variables and professional category, and the older age and Southern area of residence were both confirmed as positively correlated with a more marked improvement between post- and pre-tests. As for the professional category, compared to medical geneticists as reference, a statistically significant higher progress in the *self-assessment test* was confirmed for physicians specialized in surgery (RR = 2.5), public health (RR = 2.1), psychiatry (RR = 2.1), or nuclear medicine (RR = 2.0), and for biologists (RR = 1.9).

*Completers* made up to nine attempts on the *self-assessment* post-test (Table 3), with 67.3% making only one attempt (61.7 and 70.9% among biologists and physicians, respectively). Most *Completers* made all attempts on the same day, with an average interval of 18 min between attempts. The longest average interval was observed among those who made two attempts (28.8 min), with a slight difference between biologists (24.7 min) and physicians (32.4 min). Overall, the average score on the best attempt improved as the number of attempts increased.

**Table 3:** Performance of *Completers* in the *self-assessment* post-test.

Post-test—Number of attempts	Number	%	Average time between two consecutive attempts (in minutes)	Average post-test score (best attempt) %
Biologists				
1	82	61.7	-	59.0
2	30	22.6	24.7	81.1
3	11	8.3	13.8	84.8
4–9	10	7.5	12.8	93.3
All	133	100	17.2	68.7
Physicians				
1	151	70.9	-	67.9
2	35	16.4	32.4	83.8
3	15	7.0	14.5	86.7
4–9	12	5.6	12.2	94.4
All	213	100	18.6	73.4
All completers				
1	233	67.3	-	64.8
2	65	18.8	28.8	82.6
3	26	7.5	14.2	85.9
4–9	22	6.4	12.4	93.9
All	346	100	18.0	71.6

Number and percentage of *Completers*, average time (in minutes) between two consecutive attempts, and average percentage of correct answers (average scores) obtained in the *self-assessment* post-test (best attempt) are displayed according to the Completers’ categories (biologists vs. physicians) and number of attempts to complete the self-assessment post-test.

As to the *final certification test*, 150 *Completers* made only one attempt (~43%), 145 made two attempts (~42%) and only 51 made three attempts (~ 15%) to pass the test (Table 2). When considering the best attempts, the average final score of the *Completers* was nearly 85%, i.e., ten percentage points above the passing threshold. When considering the first attempt, the average final score was nearly 69%, i.e., 6 points below the passing threshold. The score at first attempt decreased in the three subgroups (84.2%, 60.8 and 50.2%, respectively, for participants who passed the test after one, two, and three attempts). Interestingly a similar trend was observed in the scores of *self-assessment test*. *Completers* who took the *certification test* after three attempts gained the minimum score in the pre-test (42.7%) and the maximum score (and score increase) in the post-test (67%, +24.3%). When considering the best attempt, differences by sex, age, area of residence and professional category were generally minimal and not statistically significant, likely due to the limited sample size. Discrepancies by professional category were larger at first attempt and the results confirmed a better performance for geneticists (84.8%), followed by residents, radiologists, and primary care and public health specialists. The score at first attempt was 67.3% for the biologists against an average 70.7% for all physicians. The proportion of biologists who passed the course on their first attempt was lower (*N* = 42, 32%) than that of physicians (*N* = 108, 51%) and 20% of biologists used three attempts compared to 12% of physicians (Table 4). The average time between the first and best attempt to complete the *final certification test* was 8.8 days for the entire group of *Completers*, and was slightly longer for biologists compared to physicians (9.6 vs. 8.2 days), mainly due to the difference observed among *Completers* who made three attempts to pass the test (13.7 days for biologists vs. 9.7 days for physicians).

**Table 4:** Comparison of the performance of biologists vs. physicians by number of attempts to pass the *final certification test*.

Final Certification Test – Number of attempts	Number	%	*Self-assessment test – Average scores*				*Final certification test*		
			Pre-test (single attempt)	Post-test (best attempt)	Difference (post-test vs. pre-test) (%)	*p*-value	Average scores		Average number of days between the first and best attempt
			(%)	(%)			Best attempt	First attempt	
Biologists									
1	42	32%	53	66.9	13.9	0.0008	85.4	85.4	-
2	65	49%	52.6	71	18.5	<0.0001	85.2	62	7.8
3	26	20%	46.5	66	19.6	0.0003	84.3	49.5	13.7
Total	133	38%	51.5	68.7	17.2	<0.0001	85.1	67.3	9.6
Physicians									
1	108	51%	58.9	77	18.1	<0.0001	83.7	83.7	-
2	80	38%	51.5	70.1	18.6	<0.0001	86.2	58.9	7.6
3	25	12%	38.7	68	29.3	<0.0001	85.5	51	9.7
Total	213	62%	53.7	73.4	19.6	<0.0001	84.8	70.7	8.2
All completers									
1	150	43%	57.2	74.2	17	<0.0001	84.2	84.2	-
2	145	42%	52	70.5	18.5	<0.0001	85.7	60.8	7.7
3	51	15%	42.7	67	24.3	<0.0001	84.9	50.2	11.5
Total	346	100%	52.9	71.6	18.7	<0.0001	84.9	69.4	8.8

Number and percentage of Completers, average percentage of correct answers (average scores) obtained in the *self-assessment* tests (single attempt for the pre-test and best attempt for the post-test) and in the *final certification* test (best and first attempts), and average number of days between the first and the best attempt according to the Completers’ categories (biologists vs. physicians) and to the number of attempts to complete the final certification test are shown. Absolute differences (post- vs. pre-test) and *p*-values are indicated.

We observed, on average, a statistically significant, albeit variable, improvement between post- and pre-*self-assessment* for each question of each LO (Table 5). The difference in the percentage of correct answers between post- and pre-tests ranged from 5.5% (LO4, Question Q#10) to 35.2% (LO3, Q#7). Notably, low scores in the pre-test were associated with the highest score improvements in the post-test. Questions with low average scores in the post-test may highlight the need for revision to improve the clarity of the training materials and/or of the wording (e.g., Q#5). In the differential analysis between biologists and physicians, the latter showed generally better scores in the *self-assessment* results (Table 6). The differences between the two categories, however, were not statistically significant with the exception of Q#4, the only one in which the increase in correct answers between pre- and post-tests was significantly higher for physicians (+16%) than for biologists (+3.1%).

**Table 5:** *Completers’* performance for each question of the *self-assessment test*.

LO	LO description	Question	Correct answers pre-test (single attempt)		Correct answers post-test (best attempt)		Difference of correct answers (post-test vs. pre- test)	*p*-value
			*N*	%	*N*	%	%	
1	Basic elements of genetics/genomics in oncology	1.Why cancer is a genetic disease?	241	*69.7*	300	*86.7*	17.0	<0.0001
		2.Mendelian inheritance refers to patterns by which genes and traits are passed on from parents to their children. Which are the most common patterns?	256	*74.0*	302	*87.3*	13.3	<0.0001
		3.What does the term “pathogenic variant” mean?	242	*69.9*	283	*81.8*	11.9	<0.0001
2	Genetic/genomic tests for cancer diagnosis	4.What is the primary goal of molecular genetic testing of cancer?	201	*58.1*	239	*69.1*	11.0	0.0003
		5.What is involved in mainstreaming cancer genetics?	37	*10.7*	115	*33.2*	22.5	<0.0001
		6.Which of the following statements is correct regarding cancer predisposing genes, penetrance of genes/alleles, and polygenic risk score?	130	*37.6*	218	*63.0*	25.4	<0.0001
3	Risk assessment in hereditary cancers	7.Which of the following is not a marker of inherited cancer predisposition?	76	*22.0*	198	*57.2*	35.2	<0.0001
		8.How are hereditary cancers?	243	*70.2*	306	*88.4*	18.2	<0.0001
		9.Which of the following is not a marker of Lynch syndrome?	126	*36.4*	226	*65.3*	28.9	<0.0001
4	Pharmacogenomics and personalized medicine	10.What is “personalized medicine”?	269	*77.7*	288	*83.2*	5.5	0.0393
		11.What is “pharmacogenetics”?	139	*40.2*	220	*63.6*	23.4	<0.0001
		12.What is “targeted therapy”?	235	*67.9*	277	*80.1*	12.2	<0.0001

Number and percent of correct answers scored by the *Completers* for each question of the *self-assessment tests* at the entry (pre-test, single attempt) and at the end (post-test, best attempt), identified by the corresponding Learning Objective (LO). Absolute difference between post- and pre-test average percentages of correct answers and corresponding *p*-values are shown.

**Table 6:** Completers’ performance for each question of the *self-assessment test*.

LO	Question	Biologists						Physicians						Difference biologists vs. physicians (*p*-value)
		Correct answers pre-test (single attempt)		Correct answers post-test (best attempt)		Difference (post-test vs. pre- test)	*p*-value	Correct answers pre-test (single attempt)		Correct answers post-test (best attempt)		Difference (post-test vs. pre- test)	*p*-value	
		*N*	*%*	*N*	*%*	%		*N*	*%*	*N*	*%*	%		
1. Basic elements of genetics/genomics in oncology	1.Why cancer is a genetic disease?	90	67.7	115	86.5	18.8	0.0004	151	70.9	185	86.9	16.0	<0.0001	0.6181
	2.Mendelian inheritance refers to patterns by which genes and traits are passed on from parents to their children. Which are the most common patterns?	99	74.4	111	83.5	9.1	0.0516	157	73.7	191	89.7	16.0	<0.0001	0.2033
	3.What does the term “pathogenic variant” mean?	94	70.7	113	85.0	14.3	0.0004	148	69.5	170	79.8	10.3	0.0068	0.4844
2. Genetic/genomic tests for cancer diagnosis	4.What is the primary goal of molecular genetic testing of cancer?	78	58.6	82	61.7	3.1	0.5465	123	57.7	157	73.7	16.0	<0.0001	0.0324
	5.What is involved in mainstreaming cancer genetics?	13	9.8	38	28.6	18.8	<0.0001	24	11.3	77	36.2	24.9	<0.0001	0.2662
	6.Which of the following statements is correct regarding cancer predisposing genes, penetrance of genes/alleles, and polygenic risk score?	54	40.6	81	60.9	20.3	0.0003	76	35.7	137	64.3	28.6	<0.0001	0.2224
3. Risk assessment in hereditary cancers	7.Which of the following is not a marker of inherited cancer predisposition?	26	19.5	76	57.1	37.6	<0.0001	50	23.5	122	57.3	33.8	<0.0001	0.5424
	8.How are hereditary cancers?	87	65.4	109	82.0	16.6	0.0009	156	73.2	197	92.5	19.3	<0.0001	0.6106
	9.Which of the following is not a marker of Lynch syndrome?	47	35.3	83	62.4	27.1	<0.0001	79	37.1	143	67.1	30.0	<0.0001	0.6323
4.Pharmacogenomics and personalized medicine	10.What is “personalized medicine”?	98	73.7	103	77.4	3.7	0.3841	171	80.3	185	86.9	6.6	0.0522	0.6065
	11.What is “pharmacogenetics”?	51	38.3	81	60.9	22.6	<0.0001	88	41.3	139	65.3	24.0	<0.0001	0.8304
	12.What is “targeted therapy”?	85	63.9	105	78.9	15.0	0.0032	150	70.4	172	80.8	10.4	0.0045	0.4316

Biologists (*N* = 133) vs. physicians (*N* = 213). Number and percent of correct answers scored by the Completers for each question of the *self-assessment tests* at the entry (pre-test, single attempt) and at the end (post-test, best attempt), identified by the corresponding Learning Objective (LO), are shown. Absolute difference between post- and pre-test average percentages of correct answers and corresponding *p*-values are also indicated as well as the *p*-values for the comparison of the post- vs. pre-test differences between biologists and physicians.

**Figure 3:**
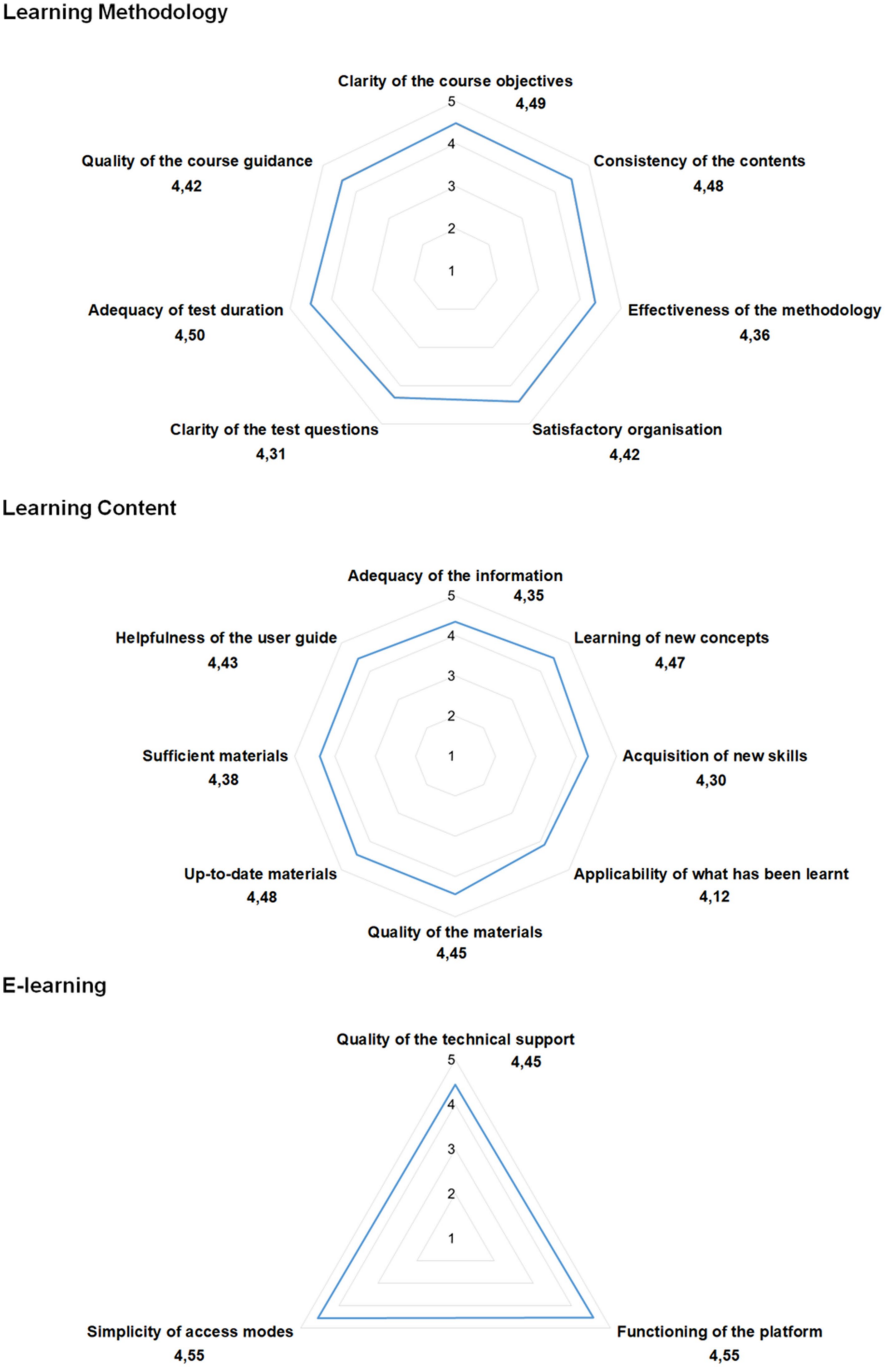
Results of the *Satisfaction Questionnaire*. Radar charts displaying the *Completers*’ responses (mean scores). The closed questions were Likert-type scale from 1 to 5 (1 = I do not agree at all; 2 = I do not agree; 3 = I neither agree nor disagree; 4 = I agree; 5 = I strongly agree).

All *Completers* filled out the *satisfaction questionnaire*, overall rating the different aspects of the course as positive or very positive (Figure 3). The average score was between 4 and 5 for each of evaluated aspects relevant to the effectiveness of the learning methodology, quality of the content, and usability of the e-learning platform. The lowest level of satisfaction expressed by the *Completers* related to the applicability of the newly learned concepts in their professional context (4.12) and to the acquisition of new skills (4.3). These results are consistent with the professional characteristics of the participants, who were not directly dealing with oncogenomics in their daily practice, and with the nature of the course (the e-learning setting is less suitable for training skills and abilities). The results obtained from the very limited non-Italian cohort of participants confirmed the same trends observed in the Italian one. Notable open comments pointed to the English language as a barrier and highlighted that specific questions/tutorials could be improved and more clinical and practical examples provided.

## Discussion

Several studies reported that non-genetic health professionals have limited knowledge of cancer genomics (13, 23–26). A properly trained health care workforce, capable of judicious interpretation, communication and translation of test results into clinical practice has become an invaluable, sought-after asset (14, 27). Investments on reorganization and improvement of education and training paths are needed to bridge the reported knowledge gaps.

Within the framework of the EU-funded JA-iPAAC (WP6), we developed and piloted the e-learning course *Oncogenomics for Health Professionals*, covering the basic elements of genetics and the OMICS technologies applied to oncology. With 346 Italian (and 28 non-Italian) *Completers* the pilot phase proved a valuable test. Italian *Completers* included physicians (the primary target) and biologists, with representative distribution of both sexes and all ages and geographic regions of residence.

In post- vs. pre-test assessments, we found an overall significant increase (~19%) in the average percent of correct answers, which is slightly higher than previously reported (28), corroborating the suitability of our methodological choices. The observed increase did not significantly vary by sex. The results suggest a greater improvement for older professionals (>50 years), who obtained lower scores at start compared to younger participants – this probably reflects the evolution of cancer genomics contents in the educational programs. In the *final certification test*, the average proportion of correct answers in the best attempt was about 85%, i.e., 10 percentage points higher than the threshold that was set to pass the test. A similar score (~84%) was reached at first attempt by 43% of the *Completers*. Analyzing data only from *Completers* rather than from all learners has several implications. First, it provides a clearer assessment of the course’s effectiveness, as *Completers* are a relatively comparable group of participants who engaged fully with the course materials and passed the final test. This allows for more reliable conclusions about the course’s ability to achieve its primary objective, i.e., improving knowledge and attitude on the fundamentals of genetics and the main applications of genomic technologies in clinical oncology. However, this approach may also introduce bias, as it excludes the participants who dropped out or failed, potentially overlooking factors that contributed to lower engagement or performance. By focusing solely on *Completers*, the analysis may miss important insights into why some participants struggled with or disengaged from the course, which could be useful for refining future iterations of the program and enhancing inclusivity.

The analysis of the course’s effectiveness revealed some differences across professional categories, although a larger number of *Completers* would be required to draw definitive conclusions. Physicians, for instance, passed the *certification test* with fewer attempts on average compared to biologists, likely due to differences in their educational backgrounds and professional experiences. Physicians typically have a strong clinical focus in their training, with practical knowledge of patient care and medical decision-making. Their familiarity in approaching the clinical setting may have contributed to their higher performance on the *final certification test*. On the other hand, the education of scientists, such as biologists, is generally more research-oriented, which may not perfectly align with the clinical applications emphasized in the course. This course was by nature interdisciplinary, with the aim of providing the different actors in the Health Service with a common literacy. In light of the differences between categories, however, the implementation of adaptive learning paths tailored to each professional group could enhance the learning experience. For example, physicians might benefit from more advanced modules that dive deeper into the application of genomic technologies in clinical decision-making, while scientists could be offered additional core competencies that link their research expertise with practical clinical scenarios. Based on these considerations, adaptive learning pathways, as suggested by McCorkell and colleagues (29) would allow for a more personalized learning experience, potentially improving outcomes across different professional categories. Such refinement could not only improve engagement and success rates for all groups of learners, but also ensure that the course remains relevant and accessible to a diversified audience of health professionals.

The design and development of this e-learning course implies a number of limitations. First, the course was not offered in the participant’s native language, which may have reduced its impact and limited the participation of a larger audience. The nearly three-year delay in reporting the results of the pilot course represents another potential limitation. However, given the primarily methodological nature of the study, the overall value of the reported results is expected to be minimally affected by such a delay. Additionally, although competencies may evolve over time, the standard set of core competencies in cancer genomics for health professionals that were used to design this course (17) remains largely valid. Third, considering the highest score from the available attempts on the T1, *self-assessment test* may result in overestimating the course’s effectiveness. In line with this consideration, 57% of the *Completers* failed the *final certification test* on their first attempt. We need to consider, however, that the awareness that three attempts are available might have influenced the learners’ approach, potentially leading to less thoughtful responses on the first try. Fourth, the survey/quiz tools were not previously validated through preliminary administration to users that reflected the target audience of this training. However, the accuracy of the questions as well as their respective answer options are safeguarded, as they were developed directly by the experts responsible for the scientific content of the course. Additionally, the structure of the questions was based on and adheres to the guidelines produced by Age.na.s., the Italian provider of Continuing Medical Education (CME), for tests used in distance learning courses. Based on the results of this pilot course, in the next course, which is currently under development, we will revise the questions included in the *self-assessment* and *final certification* tests. Questions will also undergo validity and reliability analyses, which have not been conducted on the current tools. Finally, regarding the timing, most *Completers* made multiple attempts on the *self-assessment* post-test on the same day, with an average interval of 18 min between attempts. This interval seems appropriate and sufficient for a quick review of an entire video tutorial or a few slides from different tutorials. Each tutorial lasted approximately 15 min, and after watching them, participants had the option to download the content in PDF format, allowing for a quick review of key educational contents. Additionally, Age.na.s. recently established guidelines for the timing of repeated attempts on the *final certification test*. These guidelines require e-learning participants who fail an attempt to review all the educational materials before retaking the test. This policy has been applied to all courses on our e-learning platform since 2022, and will also be implemented in the next edition of this course.

A major strength of our approach resides in the methodology utilized to develop and implement the course. First, the core competencies we used as a source were identified through a rigorous process (i.e., literature review followed by expert agreement based on a Delphi process) (17) ensuring valuable transnational applicability. Moreover, the e-learning course was implemented based on the PBL methodology, to facilitate a self-learning process that is not purely notional but adapted to the professional context of the learners. The PBL method is an educational approach aligned with andragogic principles (the science of adult learning). This method fosters self-directed learning by encouraging participants to tackle realistic problems mirroring their professional experiences (21). PBL’s effectiveness in fostering critical thinking skills, among others, and its growing popularity in healthcare education (30) made it the ideal choice for this course. Notably, PBL has been successfully adapted to e-learning environments, with various models emerging that respond to different levels of interaction between participants and facilitators (20, 28).

In addition, despite undeniable limitations (i.e., lack of interaction/exchange and required acquaintance with technological systems such as the online platform), the e-learning format offers several advantages to both the organizer and the users. For the former, the affordability of the set up and delivery and the versatile nature of the online modules ensure cost-effective dissemination to a broad audience. For the latter, flexibility in the schedule and inexpensive access to several tools are incentive factors to participation.

Based on the *satisfaction assessment*, the e-learning course was very positively evaluated, in terms of methodology and quality of the contents as well as operability of the delivery platform. International testing of the module, albeit limited, yielded results in line with what observed in Italy. This encouraged us to seek a broader reach. The feedback collected via the *satisfaction questionnaire* also highlighted challenging subjects and/or sections that needed improvement. Building on this ground, our future steps will aim at fine-tuning the e-learning course to move toward its wide implementation. Since we recognize that the language barrier and the lack of CME certification in countries other than Italy may be an obstacle to larger divulgation, we will tackle also these potential hurdles.

Our planned future steps will be made under the umbrella of the CAN.HEAL project,^3^ launched in November 2022, which strives to promote synergy between the public health and clinical dimensions of cancer genomics and includes an *Education and Training*-focused WP13. We plan to improve the e-learning course based on the participants’ feedback received during its pilot phase and to appoint an international committee for revision of the course material prior to its wider release. We will also address the identified barriers to dissemination and incentivize participation by translating the course materials into different languages and pursuing European and, where needed, country-specific CME accreditation. We will seek optimal delivery of the revised e-learning course through national distance training platforms in different countries.

As technology advances at a very fast pace, so should educational and training programs to forge a genetics/genomics literate and competent health care workforce able to stay abreast of the rapidly evolving scenario and to handle, skilfully, the vast amount of acquired information and integrate it into clinical practice. Overcoming the health literacy gaps is posed to be a challenge that needs to be addressed so that the potential of genomic medicine can be fully realized.

## Data Availability

The original contributions presented in the study are included in the article/supplementary material, further inquiries can be directed to the corresponding author.

## Footnotes

^1^ https://www.ipaac.eu/en/work-packages/wp6/

^2^ https://www.eduiss.it

^3^ https://canheal.eu/
